# A preliminary exploration of the feasibility of offering men information about potential prostate cancer treatment options before they know their biopsy results

**DOI:** 10.1186/1472-6947-13-19

**Published:** 2013-02-06

**Authors:** Steven B Zeliadt, Peggy A Hannon, Ranak B Trivedi, Laura M Bonner, Thuy T Vu, Carol Simons, Crystal A Kimmie, Elaine Y Hu, Chris Zipperer, Daniel W Lin

**Affiliations:** 1 Northwest HSR&D Center of Excellence, VA Puget Sound Health Care System, Metropolitan Park West, 1100 Olive Way #1400, Seattle, WA 98101, USA; 2 Department of Health Services, University of Washington School of Public Health, Seattle, WA, USA; 3 Seattle Institute for Biomedical and Clinical Research, Seattle, WA, USA; 4 Washington State Department of Health, Cancer Prevention and Control Unit, Olympia, WA, USA; 5 Department of Veterans Affairs Medical Center, Urology Service, Seattle, WA, USA; 6 Department of Urology, University of Washington School of Medicine, Seattle, WA, USA

**Keywords:** Prostate cancer, Biopsy, Decision aid, Prostate biopsy, Treatment decision making

## Abstract

**Background:**

A small pre-test study was conducted to ascertain potential harm and anxiety associated with distributing information about possible cancer treatment options at the time of biopsy, prior to knowledge about a definitive cancer diagnosis. Priming men about the availability of multiple options before they have a confirmed diagnosis may be an opportunity to engage patients in more informed decision-making.

**Methods:**

Men with an elevated PSA test or suspicious Digital Rectal Examination (DRE) who were referred to a urology clinic for a biopsy were randomized to receive either the clinic’s usual care (UC) biopsy instruction sheet (n = 11) or a pre-biopsy educational (ED) packet containing the biopsy instruction sheet along with a booklet about the biopsy procedure and a prostate cancer treatment decision aid originally written for newly diagnosed men that described in detail possible treatment options (n = 18).

**Results:**

A total of 62% of men who were approached agreed to be randomized, and 83% of the ED group confirmed they used the materials. Anxiety scores were similar for both groups while awaiting the biopsy procedure, with anxiety scores trending lower in the ED group: 41.2 on a prostate-specific anxiety instrument compared to 51.7 in the UC group (p = 0.13). ED participants reported better overall quality of life while awaiting biopsy compared to the UC group (76.4 vs. 48.5, p = 0.01). The small number of men in the ED group who went on to be diagnosed with cancer reported being better informed about the risks and side effects of each option compared to men diagnosed with cancer in the UC group (p = 0.07). In qualitative discussions, men generally reported they found the pre-biopsy materials to be helpful and indicated having information about possible treatment options reduced their anxiety. However, 2 of 18 men reported they did not want to think about treatment options until after they knew their biopsy results.

**Conclusions:**

In this small sample offering pre-biopsy education about potential treatment options was generally well received by patients, appeared to be beneficial to men who went on to be diagnosed, and did not appear to increase anxiety unnecessarily among those who had a negative biopsy.

## Background

Men diagnosed with early stage prostate cancer have multiple potential treatment options, with no option clearly demonstrating superiority to the others [1-3]. Ideally, newly diagnosed patients should engage in a decision-making process to select the treatment with a risk and benefit profile that is most consistent with their individual preferences. Decision aids (DA) for a variety of clinical settings have been shown to improve knowledge regarding options, reduce decisional conflict, help patients feel informed about personal values, and stimulate patients to take a more active role in participating in decision making. While DA have not been shown to consistently improve satisfaction with decision making, reduce anxiety, or lead to improved health outcomes such as general quality of life, or disease-specific quality of life [4], studies of prostate cancer DA have specifically demonstrated improved knowledge and more active participation in the treatment decision [5-8].

Although professional societies have advocated the use of DA for prostate cancer treatment decision-making [9], there remains uncertainty about how to ideally implement DA in clinical practice including the optimal format (booklet, online, DVD), ideal setting (home, patient education center, urology clinic) and timing (prior to or following the visit in which the provider provides the news of the diagnosis and begins discussing treatment options). The timing of education about treatment options may be critical as prior studies have consistently observed that anxiety about the cancer diagnosis often interferes with objective processing of information about individual risks and benefits of treatment [10,11]. Two recent studies of treatment decision making observed that many patients arrive at a treatment decision almost immediately after being diagnosed, with 65% of patients considering only a single option [12,13].

The objective of this feasibility study was to explore whether patients scheduling a biopsy, of whom approximately 25-30% would go on to be diagnosed with prostate cancer, would find it acceptable to receive information about treatment options before they knew the result of their biopsy. This small study was conducted to obtain preliminary data due to concerns from our local human subjects committee that offering men educational materials about cancer during the pre-biopsy period would cause more harm than benefit. A small amount of funding was obtained from a prostate cancer advocacy group to provide proof-of-principle evidence about the feasibility and acceptability of the timing of the educational intervention because no similar studies conducted during the pre-biopsy period could be identified in the literature.

We selected an existing DA as the educational format for providing information about available treatment options, which was written for men who had received a definitive diagnosis of cancer, not as an educational tool for men being referred to biopsy. Our primary motivation was to explore whether the pre-biopsy timing of an educational intervention was acceptable to patients to provide pilot data for a future study, and not to explicitly test the content of the out-of-context DA which was developed for patients with a confirmed prostate cancer diagnosis and has been validated in that context [14].

## Methods

### Participants

We approached 53 consecutive patients in the Department of Veterans Affairs Puget Sound urology clinic at the time they were referred for biopsy for an elevated PSA test or suspicious digital rectal exam. Patients were eligible for the study if they had no prior history of prostate cancer, were able to read and understand English, and the patient’s urologist indicated that metastatic prostate cancer was not suspected and did not sense the patient was demonstrating significant emotional distress associated with the upcoming biopsy. Patient literacy was estimated using the Rapid Estimate of Adult Literacy in Medicine (REALM) [15].

### Procedures

Patients met with a study coordinator to provide written informed consent after meeting with the urologist and deciding to schedule a biopsy. All participants were informed that although most men undergoing a biopsy would not turn out to have cancer, the study was testing an educational intervention that involves providing some men in the study with materials about prostate cancer treatment options. Patients received a $5 gift certificate for use at the VA cafeteria after completing the informed consent. The study coordinator randomized participants to take home either the educational (ED) intervention packet or the usual care (UC) packet which included the clinic’s standard biopsy instruction sheet. Both the coordinator and participant were blinded to randomization assignment until the packet was provided to the subject.

A research coordinator contacted study participants by telephone for a baseline interview 5 days after participants received the study materials. The coordinator monitored the VA’s electronic medical record to identify when the biopsy was performed and the results had been discussed with the patient. Patients were contacted for a second interview 2 weeks after confirmation that the patient had received their biopsy results. The VA Puget Sound Health Care System’s Institutional Review Committee and Research and Development office, and the Washington State Human Subjects Committee, approved all procedures.

### Educational intervention

Intervention participants received the current print version of the treatment DA developed by the Michigan Cancer Consortium entitled: “Making the Choice: Deciding What to Do About Early Stage Prostate Cancer” [16]. Because the DA is written for men who know they have cancer, we provided a cover letter explaining that the Michigan DA *“is being provided to you today, before your biopsy, so that you can look over these materials and can learn more about prostate cancer and be prepared to better understand the results of the biopsy procedure.”* Also, because the DA is tailored for men who have the results of their biopsy including tumor characteristics such as grade and stage, we developed a brief booklet describing the biopsy procedure and explained concepts including Gleason grade. The booklet was reviewed by prostate cancer advocates through the Washington State Prostate Cancer Task Force. The booklet was 12 pages, with a 7.3 Flesch-Kincaid Grade Level. The booklet is available as Additional file 1.

### Measures

We assessed utilization of the educational materials, prostate cancer knowledge, general depressive symptoms, prostate cancer specific anxiety, general quality of life, and processes of care at the baseline interview for all participants, as well as at post-biopsy follow-up. During the follow-up evaluation, men with positive biopsy results received additional cancer-related measures.

#### Utilization of the educational materials

Using an embedded mixed-methods design, study participants were asked a series of quantitative and qualitative items to assess use of the ED or UC materials and to obtain participant feedback. Participants were asked if they used the materials, how much time they spent with the materials, and if they shared the materials with anyone else. They were asked to rate on a scale from 1 to 6 how helpful the materials were in understanding prostate cancer and guiding them in making a treatment decision. Men were specifically asked to describe sections of the materials they found to be most helpful. Participants were also asked about use of the internet to search for treatment information before the biopsy at the baseline interview and men who were diagnosed with cancer were asked about internet searches at the follow-up interview. Following each interview, the interviewer recorded on a 6 point scale each subject’s general engagement in the study across 3 domains - ease of responding to the interview items, impatience with the interview, and perceived mistrust of the VA.

#### Familiarity with treatment options & knowledge

The interview included two items about familiarity with potential treatment options at the baseline interview. The first question asked patients to volunteer any treatment options they would consider if they were diagnosed: “*Although you may not have cancer, we would like to know what treatments you think you might consider if you were to have prostate cancer. Are there any treatments you have heard about that you think you would want or are there any treatments you’ve heard of that you know you do NOT want?”* The interviewer recorded any options the patient volunteered, and then asked a second question: “*There are some common treatments available that you did not mention. Do you think you might consider any of these if you were told you had prostate cancer?*” The five treatments we probed for included open surgery, robotic-assisted surgery, external beam radiation, seed radiation/brachytherapy, and active surveillance/watchful waiting. We asked 11 prostate cancer knowledge items from a prior trial of a PSA screening decision aid, selecting items relevant to the treatment context [17]. Each item counted as a correct or incorrect answer, e.g. 11 correct answers was scored as 100%.

#### Anxiety, distress & quality of life

Prostate specific anxiety was assessed using items from the Memorial Anxiety Scale for Prostate Cancer [18]. For the baseline interview we utilized the original items, with the following introduction “*Now I am going to read you a list of comments made by men about prostate cancer. These are standard measures about prostate cancer that we are asking everybody, even though you may NOT have prostate cancer. Please tell me how frequently these comments were true for you during the past week.”* General distress and mood were assessed using the Patient Health Questionnaire (PHQ-8), a validated rating scale of depression symptoms [19,20]. We also asked about general quality of life using a single item: “*Imagine a thermometer where 0 is worst possible health and 100 is best possible health. Where would you rate your overall health during the past week - between 0, worst possible health, and 100, best possible health*?”.

#### Processes of care

We utilized 16 items from the short form of Stewart’s Interpersonal Processes of Care Instrument focusing on domains relevant to the biopsy process including: clarity of communication, elicited concerns, explained results, interpersonal style, discrimination and disrespectful staff. Treatment medications and post-treatment decision making domains were excluded [21].

#### Control preferences scale

We used the single item Control Preferences Scale to assess preferences for involvement in prostate cancer treatment decision [22]. This question was phrased hypothetically at the baseline interview for all participants, asking men to imagine their preferences for participating in the decision process *if* they were to be diagnosed with cancer.

#### Cancer related measures

The follow-up interview for patients diagnosed with cancer was tailored to include additional measures about the patient’s treatment decision-making experience and adjustment to cancer. These men were asked the 10-item version of the Decisional Conflict Scale [23], and we assessed involvement in the decision process using five items from the Assessment of Patients’ Experience of Cancer Care (APECC) study [24,25].

### Qualitative analysis

Descriptive analyses summarized the findings of the semi-structured interview items assessing use of the materials. Participants were coded as using the materials only if they were able to clearly articulate which sections in the materials they found to be helpful. Two reviewers (SZ&LB) independently reviewed the responses to open-ended baseline and follow-up interview, including the question “*Would you have preferred it if we had NOT given these types of materials out to men prior to the biopsy?*” Then SZ & LB discussed emerging themes and selected exemplary quotations to highlight.

### Data analyses

Exploratory analyses were conducted comparing outcomes between the ED and UC groups using chi-square tests to compare categorical variables and t tests to compare continuous instrument scores. For instruments in which there was a single missing response for one of the items, we imputed the value based on the mean of the remaining items. Imputations were performed for two participants for the Process of Care measure, two participants for the Memorial Anxiety Scale, and one subject for the abbreviated Mini-Mental Adjustment to Cancer scale. All quantitative analyses were conducted as intent-to-treat regardless of whether the subject reported using the educational materials. Due to the small sample size no multivariate comparisons were conducted. All analyses were conducted using Stata 11.0 (StataCorp, College Station, TX).

## Results

### Willingness to participate

**Figure 1:**
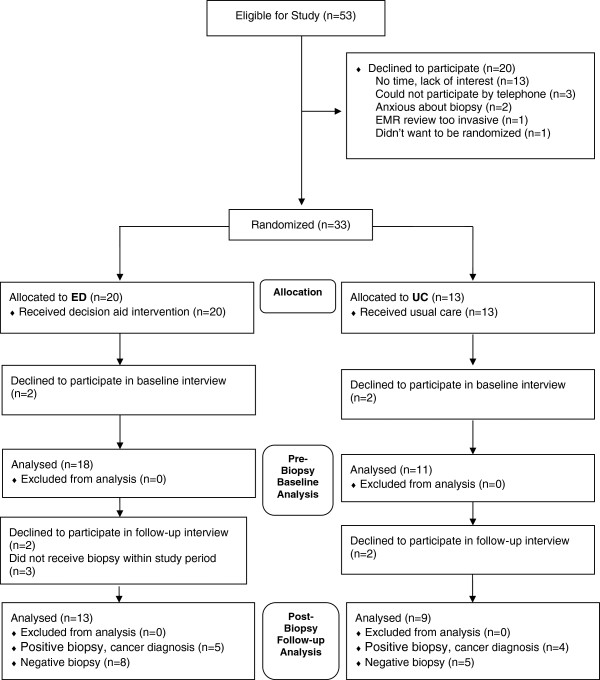
Study flow.

We approached 53 potential study participants, of whom 33 (62%) agreed to enroll (Figure 1). Two men declined to enroll because they were anxious, 3 declined because they didn’t have a telephone or didn’t like the idea of talking on the phone for the interviews, one indicated he didn’t want the study investigators looking at personal information in the electronic medical record, one indicated he didn’t like the idea of randomization, and the remaining 13 indicated they didn’t have the time or weren’t interested in participating in a study. Four enrolled participants (2 from each arm) declined to complete an interview when called. Our final sample included 29 men completing baseline interviews – 18 men who received the ED materials and 11 men who received UC materials. We were able to conduct follow-up interviews with 22 men (76%) after they received their biopsy results. Among men completing the follow-up interview, 13 had received ED materials and 9 had received UC materials. Five (39%) of the ED participants were diagnosed with prostate cancer while four (44%) of the UC participants were diagnosed with cancer. Patient demographics were similar across the ED and UC groups (Table 1).

**Table 1:** Demographic and clinical characteristics

	**Usual care (n = 11)**	**Education intervention (n = 18)**	**P-value**
Mean age, years (s.d)	61.8 (6.5)	64.2 (5.8)	0.32
White, n (%)	10 (91%)	17 (94%)	0.71
Currently married, n (%)	6 (55%)	10 (56%)	0.95
College or graduate degree, n (%)	3 (27%)	5 (28%)	0.97
REALM reading score, mean (s.d)	64.3 (2.0)	64.3 (2.6)	0.99
Completed follow-up interview, n (%)	9 (82%)	13 (72%)	0.55
Positive biopsy result, n (%)	4 (44%)	5 (38%)	0.77

### Use of the educational materials

The ED intervention was well received (Table 2). At the baseline telephone interview within a few days of receiving the materials, 83% of ED participants reported having read the materials compared to 46% of UC participants (p = 0.03). A large portion of the ED group (78%) indicated the materials were useful and were able to refer to a specific section of the materials during the interview compared to only 36% of UC participants (p = 0.02). There were no significant differences between groups on impatience during the interview, ease of responding during the interview, or expressions of mistrust of the VA. The interviewer (CS) rated 7 ED participants as “very engaged” in the interview with the remaining as “somewhat” engaged. All of the UC participants were rated as “somewhat engaged”.

**Table 2:** Comparison of baseline (pre-biopsy) outcomes between usual care and education intervention groups

	**Usual care (n = 11)**	**Education intervention (n = 18)**	**P-value**
**Utilization of the study materials**			
Yes, read the materials, n (%)	5 (45%)	15 (83%)	0.03
Specified something was especially helpful, n (%)	4 (36%)	14 (78%)	0.02
Materials were very or extremely helpful in understanding prostate cancer, n (%)	2 (18%)	10 (56%)	0.04
Patient/family went to internet for information before biopsy, n (%)	2 (18%)	8 (44%)	0.14
**Familiarity with treatment options & prostate cancer knowledge**			
Number of treatments patient is familiar with out of 4 main options, mean (s.d.)	3.0 (1.4)	3.5 (1.0)	0.28
Number of men familiar with watchful waiting/active surveillance, n (%)	5 (46%)	11 (61%)	0.67
Percent of knowledge items answered correctly, mean (s.d.)	33% (25)	42% (23)	0.32
**Anxiety & distress**			
Memorial anxiety scale, mean (s.d.) scored 0-100; higher = more anxiety	51.7 (20.7)	41.2 (15.8)	0.13
Patient health questionnaire (PHQ8), mean (s.d.) scored 0-24; higher = more depressive symptoms	9 (6.8)	7.2 (6.9)	0.52
Overall quality of life, mean (s.d.) Scored 0-100; higher = better quality of life	48.5 (30.6)	76.4 (18.2)	<0.01

When asked “*Would you have preferred it if we had NOT given these types of materials out to men prior to the biopsy?*” at the baseline interview, everyone indicated the materials should be provided, and elaborations such as the following were typical: “I think everyone should get it, not knowing raises anxiety.” However, in response to the same question during the second interview we obtained more mixed opinions. One ED participant described distributing the intervention prior to biopsy as “trying to fix a problem before it exists” and another recommended that distribution of educational materials be left up “to the individual patient”. Both of these participants had been diagnosed with prostate cancer upon biopsy. The majority of participants at the follow-up interview continued to express generally positive opinions of the ED, with statements such as “They are very helpful, necessary and should be given out”. A UC subject who had been diagnosed with cancer remarked, “I would have liked to have more info on what prostate cancer is and its treatment before I got the results. It was a shock.” An ED subject who had been diagnosed with cancer stated, “I highly recommend everyone have access to all the educational material…it reduces anxiety”.

### Familiarity with treatment options and knowledge

There was some association with increased knowledge about prostate cancer treatments with the ED, as we observed a 15 percentage point increase in the number of men receiving the ED materials who were familiar with watchful waiting/active surveillance (p = 0.67) at baseline and a 10 percentage point improvement in baseline accuracy of knowledge items (p = 0.32) compared to the UC group, although neither was statistically significant in this small sample (Table 2). However, we saw few differences in knowledge at the follow-up interview among men diagnosed with cancer as both the ED and UC groups were able to answer the same number of knowledge items correctly (57% vs. 59%).

### Anxiety, distress and quality of life

The ED intervention did not appear to increase anxiety at the baseline evaluation, and in fact we observed a trend toward decreased anxiety among men receiving the ED materials (Table 2). Scores from the Memorial Anxiety Prostate Cancer (MAX-PC) instrument, which was focused on worry about prostate cancer, were 10 points lower among men in the ED group compared to the UC group (41.1 vs. 51.6, p = 0.13). Men receiving the ED materials scored 2 points lower on the Patient Health Questionnaire (PHQ8) (p = 0.52). Overall quality of life assessed at baseline through a single item self-assessment rating scale was significantly better among the ED group (75.8 vs 48.5, p < 0.01).

**Figure 2:**
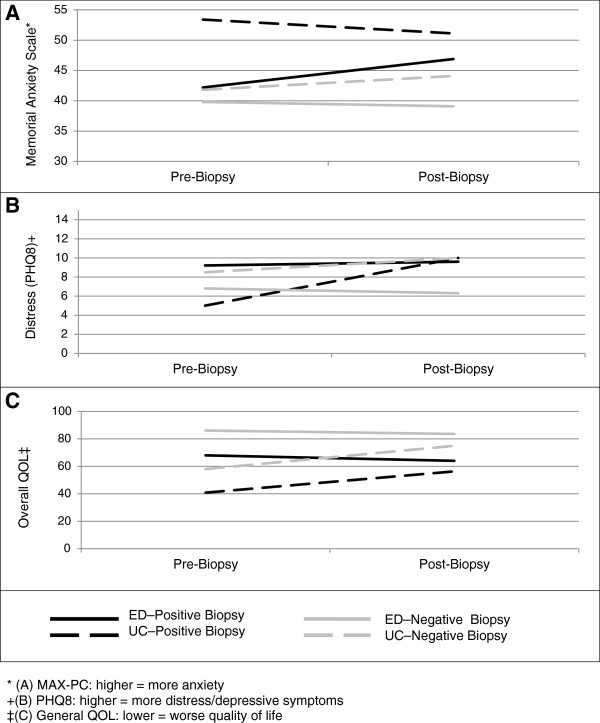
Outcomes pre- and post-biopsy: (A) Memorial anxiety scale –prostate cancer (MAX-PC); (B) Distress – patient health questionnaire 8 (PHQ8); (C) General quality of life (QOL).

At the follow-up interview we continued to see lower anxiety and distress scores, and improved overall self-assessed quality of life among ED participants (Figure 2). There were no significant differences between the ED group compared to the UC group with respect to lower prostate cancer anxiety scores (46.9 vs 51.5, p = 0.64) or frequency of fewer depressive symptoms (9.6 vs 10.1, p = 0.93). Unexpectedly, both prostate cancer worry and depressive symptoms were slightly higher at follow-up than at baseline for participants receiving UC materials among the participants with negative biopsy results. In contrast, in the ED group prostate cancer anxiety and depressive symptoms declined between baseline and follow-up for men with negative biopsy results (Figure 2), although differences were minor and were not statistically significant at either baseline or follow-up. The overall quality of life score remained slightly higher for the ED (83.6 vs 75.0, p = 0.13) after receipt of negative biopsy result, although the result was not significant.

### Control preferences scale

Among men diagnosed with cancer, ED and UC groups had similar preferences as to how active a role they preferred to take in the decision-making process with their provider (2.60 vs. 2.25, p = 0.36).

### Decisional conflict scale (DCS)

Men with cancer in the ED group had similar decisional conflict scores compared to UC participants (1.40 vs. 2.13, p = 0.53). We did observe a trend in the proportion of men reporting knowledge about risks of side effects in response to specific items of the DCS. All men in the ED group who were diagnosed with cancer indicated they “knew the risks and side effects of each option” and “were clear about which risks and side effects mattered most to you”. In contrast, only half of the UC group responded that they knew the risks and side effects (p = 0.07) or were clear about which side effects personally mattered most (p = 0.07).

### Processes of care

We included two sets of measures to explore whether providing education about treatment options at the time of biopsy would help patients better communicate with their physician if the results of their biopsy were positive. The intervention did not appear to have any measurable influence on facilitating patient-physician communication. Patients diagnosed with cancer in the ED group reported similar scores on the 5 items that measured physician encouragement in the decision process compared to the UC group (60.0 vs. 56.0, p = 0.90).

## Discussion

Providing information about potential prostate cancer treatment options to men preparing to undergo a prostate cancer biopsy was well received by patients, and did not appear to increase anxiety. This finding is consistent with prior evaluations of DA among newly diagnosed men which have consistently shown that DA do not increase anxiety [26]. However, to our knowledge this is the first study administering education about cancer treatment options to patients before they are definitively diagnosed with cancer. Undergoing a biopsy is stressful [27] and there are concerns that providing information about cancer treatment options to men who have not yet been diagnosed may be inappropriate. However, the biopsy process may also be a unique chance to engage men in preparing for the treatment decision-making process and provide a better opportunity to process nuanced information about the risks and benefits of treatment before they have to psychologically cope with a cancer diagnosis [28]. Our finding that providing information to patients at this unique timepoint did not increase anxiety, but rather seemed to lower anxiety and improve overall quality of life, provides strong preliminary support for engaging patients about potential treatment options early in the biopsy process.

Men receiving the educational intervention exhibited increased knowledge, although the difference did not reach statistical significance. In addition to general knowledge about risks and benefits of prostate cancer treatment, men who received the intervention reported being more familiar with available treatment options, including active surveillance. However, the gains in knowledge were only observed shortly after receiving the intervention. By the time men were diagnosed with cancer, knowledge scores and familiarity with treatment options was nearly identical between the ED and UC groups. One unexpected finding was that the intervention prompted some men to use the internet to learn more about prostate cancer. This may be because the intervention materials provided the website URLs for additional resources or the materials may have raised additional questions that men wanted to learn more about.

Although the sample size is small, every patient who received the intervention and went on to be diagnosed with cancer indicated they were “clear about the risks and side effects of each treatment option” and that they were “clear of which risks and side effects matter most to you,” compared to only half of participants in the UC group who were diagnosed with cancer (p = 0.07). These two items from the Decisional Conflict Scale highlight one of the key dimensions of prostate cancer treatment decision making – that each of the treatment options differ in its side effect profile [1]. While there continues to be uncertainty about how best to measure outcomes of decision support interventions [29], this trend may indicate that the ED seemed to increase patient familiarity with side effects of prostate cancer treatments. One prior study of prostate cancer DA has examined decisional conflict, which was a trial among newly diagnosed men comparing a generic video about prostate cancer treatment options or combining the video with a computer program to identify tailored information preferences to provide to patients [5,8]. This study did not observe a difference in decisional conflict between the two groups (p = 0.40), noting that decisional conflict was low in both groups at baseline.

Our feasibility study has several limitations. First, our study was small, with a total of 29 men participating in the baseline evaluation. Only 9 participants diagnosed with cancer and 13 with negative biopsies participated in the follow-up evaluation. Although all measures appeared to favor the ED intervention, this small sample size precludes making any firm conclusions. Although p-values are provided, they should be interpreted with caution as this study was not powered to test specific hypotheses about the ED. Second, our study was conducted exclusively within a single VA urology clinic and our findings may not be generalizable to other settings. Third, although randomization was blinded, the remaining activities of the study were not. Men were aware of which study group they were assigned, and although the study interviewer was not specifically told which group the participants had been assigned, during the interview participants were asked to describe the study materials, effectively un-blinding the interviewer. Another limitation is that we explored multiple instruments, many of which were developed for patients with cancer, which was different from the context of our study. We acknowledge that the psychometric properties of the instruments in this context have not been evaluated.

## Conclusions

Overall, this feasibility study provides strong encouragement that pre-biopsy implementation of an educational intervention about potential treatment options (ED) may be an ideal opportunity for engaging and preparing men to make informed prostate cancer treatment decisions, with little risk of harm due to increased anxiety. Patients appeared to be enthusiastic about receiving educational materials at this early stage in the process, over 80% reported using the materials, and 100% of men who went on to be diagnosed with cancer who received the educational materials reported knowing the risks and benefits of all available options and knowing which side effects mattered most to them.

## Competing interests

None of the authors have any financial or non-financial competing interests (political, personal, religious, ideological, academic, intellectual, commercial or any other) to declare in relation to this manuscript.

## Authors’ contributions

SBZ is the guarantor of the manuscript and takes responsibility for the integrity of the work as a whole from inception to published article. SBZ contributed to study design, funding, data acquisition and collection, data analysis and interpretation, and writing and revision of the manuscript. PAH contributed to study design, data analysis and interpretation, and writing and revision of the manuscript. RBT contributed to study design, data analysis and interpretation, and writing and revision of the manuscript. LMB contributed to funding, data acquisition and collection, data analysis and interpretation, and writing and revision of the manuscript. TTV contributed to study design, data analysis and interpretation, and writing and revision of the manuscript. CS contributed to study design, data analysis and interpretation, and writing and revision of the manuscript. CK contributed to study design, data acquisition and collection, data analysis and interpretation, and writing and revision of the manuscript. EH contributed to study design, data acquisition and collection, data analysis and interpretation, and writing and revision of the manuscript. CZ contributed to study design, data analysis and interpretation, and writing and revision of the manuscript. DWL contributed to study design, funding, data acquisition and collection, data analysis and interpretation, and writing and revision of the manuscript. All authors read and approved the final manuscript.

## Pre-publication history

The pre-publication history for this paper can be accessed here:

http://www.biomedcentral.com/1472-6947/13/19/prepub

## Supplementary Material

Additional file 1 Questions & answers about your prostate. Click here for file
