# Supplementary material for: A preliminary exploration of the feasibility of offering men information about potential prostate cancer treatment options before they know their biopsy results
Source: BMC Med Inform Decis Mak. 2013 Feb 6;13:19. doi: 10.1186/1472-6947-13-19 (PMC3599913; doi:10.1186/1472-6947-13-19)
Supplement: Additional file 1 — Questions & answers about your prostate. [file 1472-6947-13-19-S1.pdf]

Questions & Answers About Your Prostate

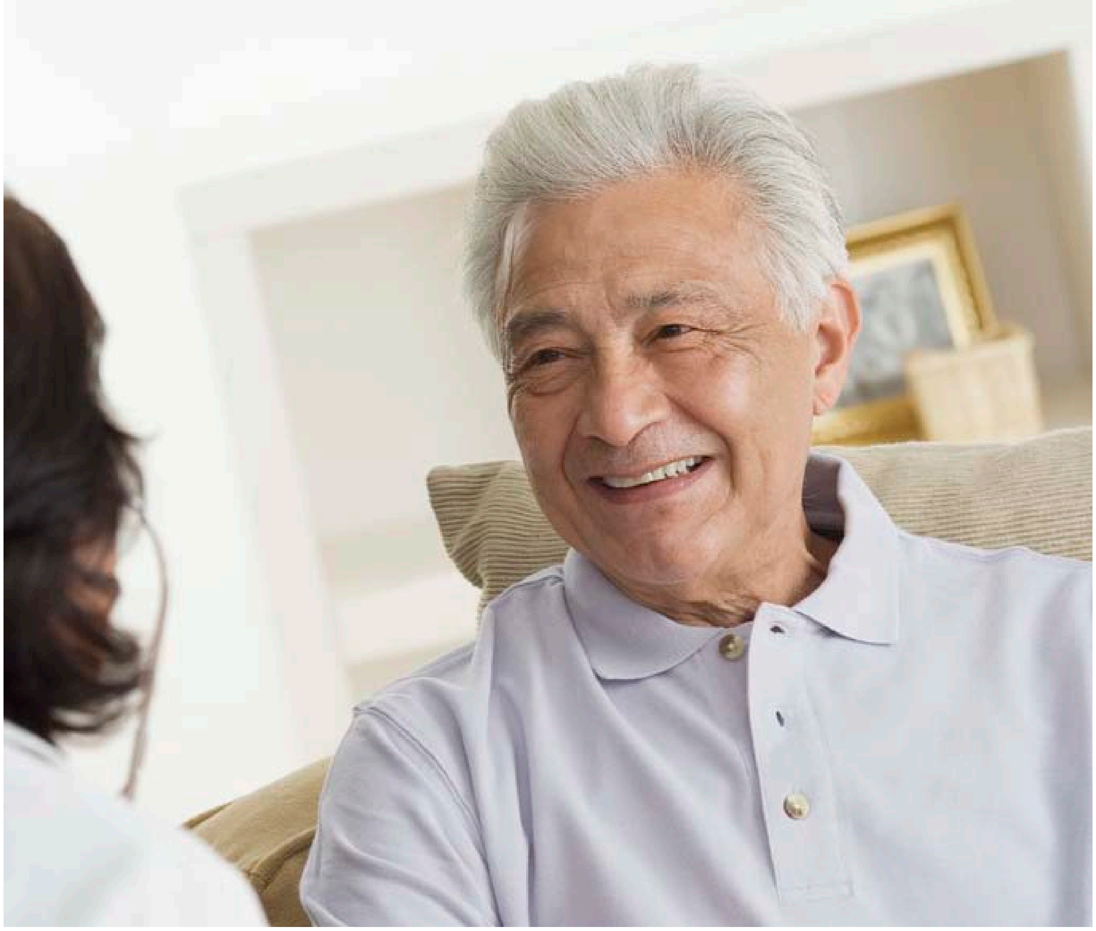

Having a biopsy test to find out if you may have prostate cancer can bring up a lot of questions. This booklet will help answer those questions.

Department of Veterans Affairs
Puget Sound
Health Care System
American Lake & Seattle

CONTENTS: WHAT'S IN THIS BOOKLET

Page

Understanding your Prostate
What it is & what it does………………..… 3
Prostate Specific Antigen (PSA)……… 3
Where it is……………………………..… 4
What can happen to it…………………..… 5

The Prostate Biopsy Test
What is a biopsy……………………….… 6
The day of the test…………………….… 7
Your chances of finding cancer……….. 8
What your test results mean.…….……... 9

Prostate Cancer
How prostate cancer is different……… 10
Choices………………………………….. 11

Where to Go for More Information........... 13

Credits………………............................... 14

This booklet is designed to help you understand medical facts and to talk with your doctor. It is not medical advice. It should not take the place of your doctor's advice and suggestions. Talk with your doctor about any questions you have.

UNDERSTANDING YOUR PROSTATE

What it is

The prostate is one of the male sex glands. It produces fluid to help transport and nourish sperm. When it is healthy, it is about the size and shape of a walnut.

What it does

When a man has sex, some fluid from the prostate mixes with the sperm made in the testes. Then the fluid (semen) gets squeezed out through the penis.

PSA – Prostate Specific Antigen

The prostate makes another substance important to you right now called **PSA** (**P**rostate **S**pecific **A**ntigen). Doctors measure the amount of PSA through a blood test.

PSA can be higher than normal in men with prostate cancer as well as in men with some other prostate conditions. These other conditions include prostate enlargement or inflammation.

UNDERSTANDING YOUR PROSTATE

Where it is

The prostate is located inside the body at the base of the penis. The prostate is located close to other organs including the bladder. The tube that carries urine from the bladder out of the penis (called the urethra) runs through the middle of the prostate. An enlarged prostate can press on this tube and make it hard for men to pee.

Doctors can feel some of the prostate by inserting a gloved finger into the rectum and touching the prostate. Your doctor may suggest a biopsy if he or she feels a lump or areas that are hard.

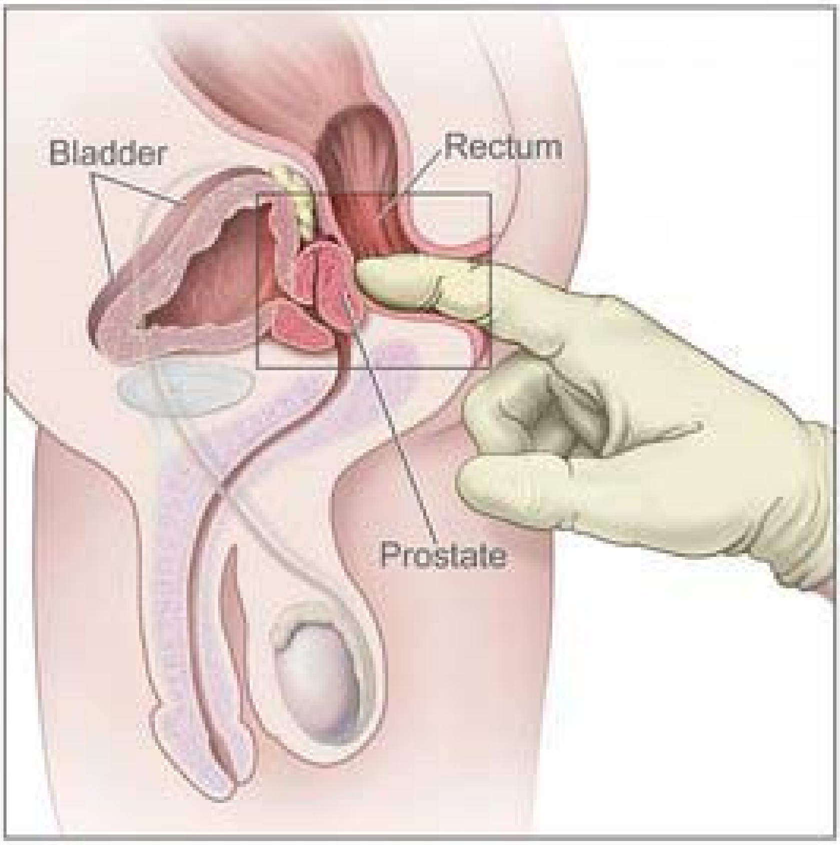

What Can Happen to Your Prostate

Enlarged Prostate or **BPH** (**B**enign **P**rostate **H**yperplasia): As you get older, the prostate can grow. If the prostate gets too large, it can put pressure on the tube inside the prostate (urethra) that carries urine and semen out of the penis. This is one of the most common causes of problems with urination among men. A wide variety of treatments are available to help with symptoms of an enlarged prostate if it becomes bothersome. Prostate enlargement is not related to the development of prostate cancer. BPH is one reason your PSA level may be high.

Prostatitis: The prostate can become tender and inflamed if irritated, or if you have an infection in the area of the bladder or prostate. The main treatments for prostatitis are usually antibiotics and pain relievers. Prostatitis is not related to the development of prostate cancer. An infection can cause your PSA level to be high temporarily.

Prostate Cancer: The prostate can also develop cancer. This is when the cells begin to grow more rapidly than normal. Prostate cancer usually doesn't produce any noticeable symptoms in its early stages. If men live long enough, most will have cancer cells in their prostate – but few will die of it. About 60 out of 100 men (60%) have cancer cells in their prostate once they reach age 70 whether they know it or not.

THE PROSTATE BIOPSY TEST

What is a Biopsy

A biopsy is a procedure in which a sample of body tissue is removed and then looked at under a microscope. The doctor will quickly insert a needle through the wall of the rectum into the prostate to remove a small sample of prostate tissue. The doctor will use a camera on an ultrasound probe to guide the needle.

Though the procedure sounds painful, it may only cause a very brief, uncomfortable sensation because it is done with a special spring-loaded biopsy instrument. The device inserts and removes the needles in a fraction of a second. Most doctors who do the biopsy will numb the area first with local anesthetic.

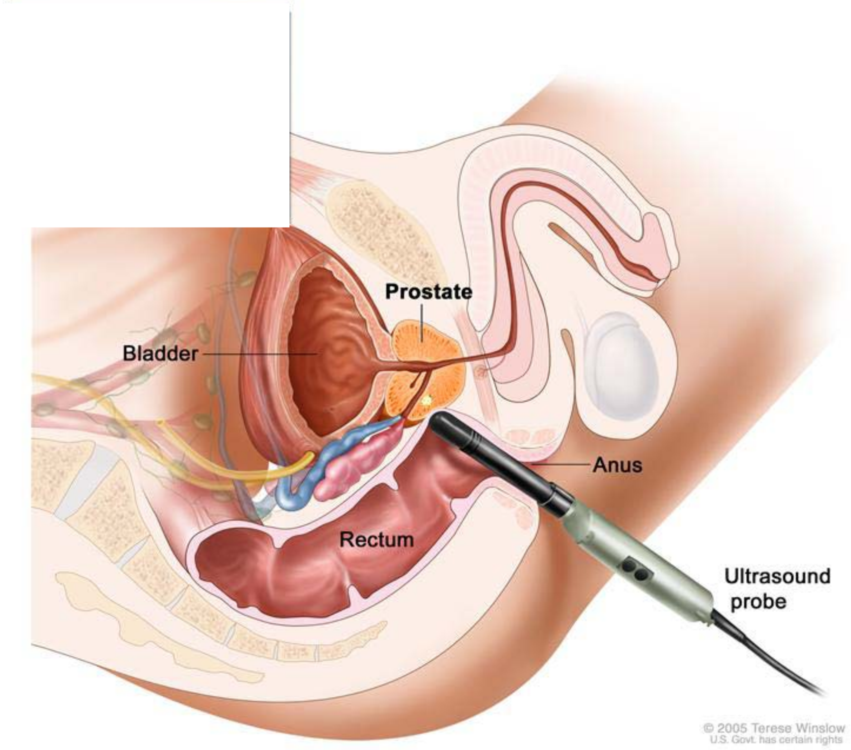

THE PROSTATE BIOPSY TEST

The Day of the Biopsy Test

The biopsy itself takes about 15 minutes and is usually done in the doctor's office.

You need to arrive 1 hour prior to your appointment time so nurses can give your antibiotic injections before the procedure. These are to help reduce the risk of infection. You will also need to take a Fleets enema about an hour before the test, or in the morning before you come to the hospital.

You may eat as you normally would any morning.

You need to stop taking blood thinning medications such as aspirin or Coumadin/warfarin several days prior to the biopsy. Please see the sheet your doctor provided about these types of medications.

You may take all of your normal medications, unless your doctor has told you otherwise.

After the Biopsy

For a few days after the procedure, you may feel some soreness in the area and will likely notice blood in your urine. You may also have some light bleeding from your rectum. Many men also see some blood in their semen and urine, which can last for several weeks after the biopsy.

A few men may develop an infection because of the biopsy. If you develop a fever greater than 101.5 degrees, you should call your doctor right away and you may need to come to the emergency room for tests.

THE PROSTATE BIOPSY TEST

Your Chances of Finding Cancer

For men with elevated PSA, the chances of finding cancer on the biopsy will vary by age, race and family history. You should discuss your chances with your doctor. In general, the biopsy will find cancer in fewer than 25% of patients getting the test.

No Cancer:	About 76 out of 100
Prostate Cancer:	About 24 out of 100

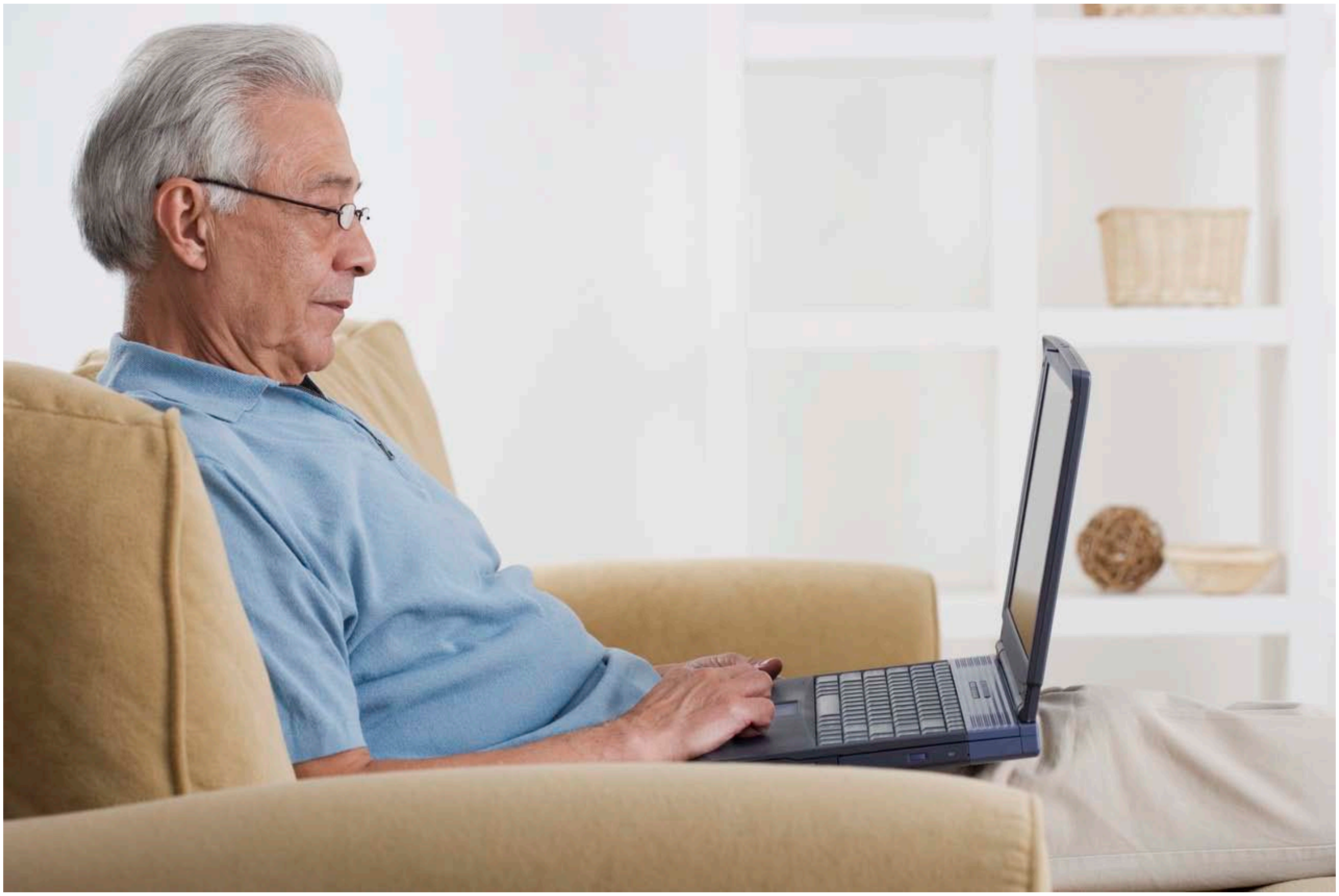

THE PROSTATE BIOPSY TEST

What Your Test Results Mean

The tissue samples are sent to the lab to see if cancer cells are present. In the lab a doctor trained to look for cancer – a pathologist - will examine the cells under a microscope.

If the pathologist finds cancer, he or she will provide a report describing the cancer. *If there is cancer*, this report will contain information about the cancer. Your doctor will discuss the findings of this report with you.

Cancer stage is how big the cancer is and whether it has spread to any areas outside of the prostate. Prostate cancer that has spread outside of the prostate to other areas of the body is called **metastatic cancer.** This is very rare for prostate cancer. Your doctors may need to do some additional tests if they think you have a metastatic cancer in order to plan your treatment.

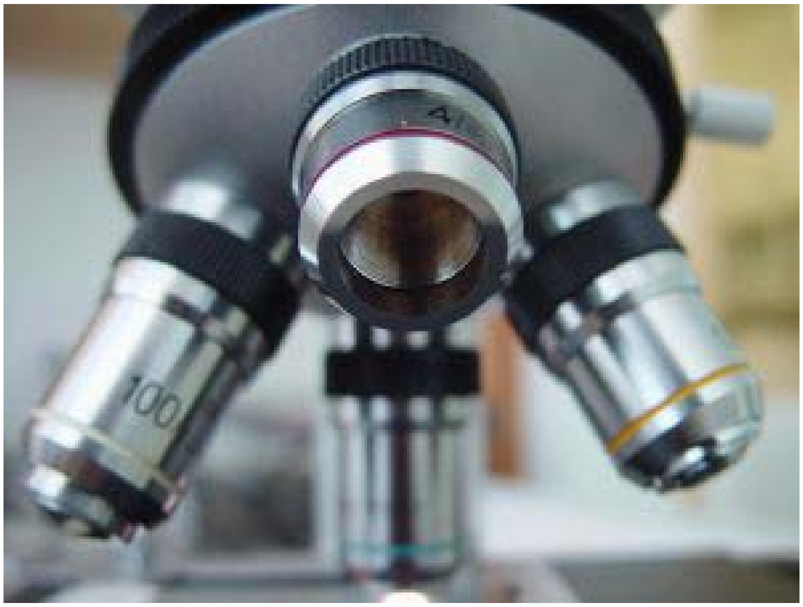

IS ALL PROSTATE CANCER BAD?

How Prostate Cancer is Different

On one hand, it is *cancer*. So, like other cancers, there's a chance it could grow and spread and even cause death.

On the other hand, prostate cancer is a very *different* kind of cancer. Prostate cancer usually grows slowly and remains inside the prostate, where it may not cause serious harm. Some prostate cancer grows slowly and may need minimal or no treatment. A few are aggressive and grow quickly.

If I Have Cancer, How Can I Tell if it is the Slow Growing Type or the Aggressive Type?

Your biopsy test is important because it will provide doctors with additional information if you do happen to have cancer. As part of the test, your doctors will closely examine any cancer cells that they find. This part of the biopsy is called the **cancer grade or Gleason Score.** This part of the biopsy test is based on how different the cancer cells are from normal prostate cells. These results can help the doctor tell if the cancer is fast or slow growing. Cancer cells may vary in size and shape. Some cells may be aggressive while others aren't.

If your biopsy test finds cancer cells, your doctor will talk to you about what type they find. There is a good chance that if you do have cancer that it is the type that is slow growing. These types of cancers are unlikely to be harmful right away, if ever. Your doctor may talk to you about watching this type of cancer for a while rather than choosing a treatment right away.

MANY OPTIONS TO TREAT PROSTATE CANCER

Choices

If the biopsy test finds prostate cancer, there are several treatments to consider including watchful waiting, surgery, or radiation. It is not easy to choose the best treatment and even the doctors don't always agree. Although you don't know yet *if you have cancer*, now may be a good time to understand some of the issues about prostate cancer treatment so that if your biopsy finds cancer you can be prepared. There is a list of resources at the end of this booklet on page 13 and your doctor will provide you with more information if your biopsy finds cancer.

What Do the Research Studies Say?

Two large studies that took more than 15 years to complete have provided new information about the number of lives potentially saved by prostate cancer screening and treatment, as well as information about potential harms. The research found that only 1 out of 48 men who received treatment had their life extended. The other 47 men were diagnosed with cancer and received treatment, but the treatment did not appear to change the course of the cancer and did not extend their life.

These studies have raised the question of whether prostate cancer screening and treatment does more harm than good. Some men may be willing to risk the potential harms of treatment, while others may prefer to avoid potential side effects of surgery and radiation treatments. Our recommendation is for you to get as much information as you can and talk to your doctor about the risks of both prostate cancer and treatment if your biopsy finds cancer cells.

Get As Much Information and Support as You Need

- Talk to your doctor and other health care providers about treatment options.
- Talk to your partner and family, and other people you trust.
- Talk to other men who have had prostate cancer.

Don't Be Pressured. Read. Ask Questions.
Think. Then Decide.

- Prostate cancer is slow growing. Even if the biopsy test finds cancer you can take your time to decide on a treatment. The 5-year survival rate for early stage prostate cancer is close to 100%, with very few men having a cancer that grows fast enough to cause death in the first few years after diagnosis.
- Each man is different. There is no right or wrong decision.
- Try to make the decision that is best for *you* – one that you can live with.

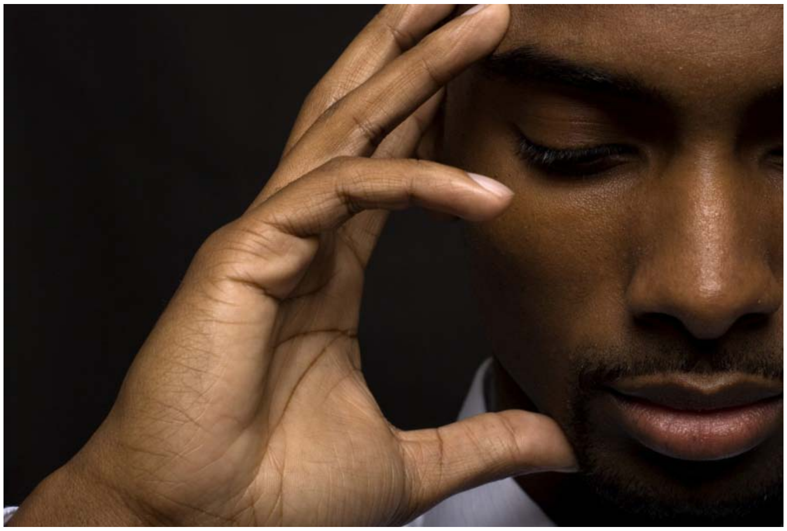

MORE INFORMATION

Michigan Cancer Consortium
Making the Choice: Deciding What to Do About Early Stage Prostate Cancer
www.prostatecancerdecision.org
1-800-249-0314

American Cancer Society
www.cancer.org
1-800-ACS-2345

US TOO – Local & National Support Groups for Prostate Cancer Survivors
www.ustoo.org
1-800-808-7866

Centers for Disease Control and Prevention (CD)
www.cdc.gov

National Cancer Institute's Cancer Information Service
www.nci.nih.gov
1-800-4-CANCER

National Comprehensive Cancer Network
www.nccn.com

Credits

Department of Veterans Affairs
Puget Sound
Health Care System
American Lake & Seattle

Alliance for Reducing Cancer Northwest
University of Washington School of Public Health

WASHINGTON
Comprehensive Cancer Control
PARTNERSHIP

WCCCP Prostate Cancer Task Force

This publication was supported by the Washington State Department of Health (Contract #RGA N17493), and by the Centers for Disease Control and Prevention (Cooperative Agreements #U58 DP000793 and #U48 DP000050)
